# Toxicity of Gutkha, a Smokeless Tobacco Product Gone Global: Is There More to the Toxicity than Nicotine?

**DOI:** 10.3390/ijerph110100919

**Published:** 2014-01-09

**Authors:** Daniel N. Willis, Mary A. Popovech, Francesca Gany, Carol Hoffman, Jason L. Blum, Judith T. Zelikoff

**Affiliations:** 1 Department of Environmental Medicine, NYU School of Medicine, 57 Old Forge Rd., Tuxedo, NY 10987, USA; E-Mails: Daniel.willis@nyumc.org (D.N.W.); marusia.popovech@gmail.com (M.A.P.); carol.hoffman@nyumc.org (C.H.); jason.blum@nyumc.org (J.L.B.); 2 Memorial Sloan Kettering Cancer Center, 1275 York Avenue, New York, NY 10065, USA; E-Mail: ganyf@mskcc.org

**Keywords:** smokeless tobacco, gutkha, nicotine, testosterone, systemic toxicity

## Abstract

The popularity of smokeless tobacco (ST) is growing rapidly and its prevalence of use is rising globally. Consumption of Gutkha, an addictive form of ST, is particularly common amongst South Asian communities throughout the World. This includes within the US, following large-scale immigration into the country. However, there exists a lack of knowledge concerning these alternative tobacco products. To this end, a study was carried out to determine the toxicity of gutkha, and what role, if any, nicotine contributes to the effects. Adult male mice were treated daily for 3-week (5 day/week, once/day), via the oral mucosa, with equal volumes (50 μL) of either sterile water (control), a solution of nicotine dissolved in water (0.24 mg of nicotine), or a solution of lyophilized guthka dissolved in water (21 mg lyophilized gutkha). Serum cotinine, measured weekly, was 36 and 48 ng/mL in gutkha- and nicotine-treated mice, respectively. Results demonstrated that exposure to nicotine and gutkha reduced heart weight, while exposure to gutkha, but not nicotine, decreased liver weight, body weight, and serum testosterone levels (compared to controls). These findings suggest that short-term guhtka use adversely impacts growth and circulating testosterone levels, and that gutkha toxicity may be driven by components other than nicotine. As use of guthka increases worldwide, future studies are needed to further delineate toxicological implications such that appropriate policy decisions can be made.

## 1. Introduction

It has been estimated that tobacco use will result in the death of one billion people worldwide in this century, and while a large majority will be due to use of cigarettes, use of tobacco in other forms also contributes to the projected outcome [1]. The use of smokeless tobacco (ST) is growing in popularity due to unsupported perception of safety, indoor smoking bans, ability to conceal use, increased social acceptance, and reported “positive” physiological effects, such as relaxation, increased concentration, heightened alertness, and diminished hunger [1]. Over the last several years, tobacco use and the ST marketplace in the United States (US) has changed dramatically with the introduction of new ST products, some of which are the result of extensive immigration from South Asia and the Middle East [2].

Smokeless tobacco is a broad term that encompasses many different types of tobacco products used both orally and nasally. Several forms of ST are linked geographically to countries around the world. The two main forms of ST used in the US are chewing tobacco and snuff [3]. Chewing tobacco comes as loose leaf, plugs, or twists, while snuff is finely ground tobacco usually placed between the gum and cheek. Prevalence of ST use by men in the US varies by state, reaching a peak of 17% in west Virginia [4]. Among US high school students, ST continues to be the most prevalent form of tobacco usage behind cigarette and cigar smoking, with 7.3% of students reporting current use [5]. 

Gutkha, also known as pan masala containing tobacco, is used primarily on the Indian subcontinent and in other regions of South Asia [6]. In addition to tobacco, gutkha, which is placed between the gum and cheek and sucked or chewed [7], contains a mixture of areca nut (seed of areca palm), slaked lime (calcium hydroxide paste), catechu (extract from the wood of the acacia plant), and a number of spices [8]. Areca nut is a known human carcinogen [9], while little is known about the toxicological implications of slaked lime or catechu. Within many regions of South Asia, the prevalence of ST use is growing rapidly, with large numbers of children and adolescents using these products [10]. In India, he prevalence of ST use is estimated at 33% for men and 18% for women [11]; that in a country with a population of over one billion [12]. Use of culturally-specific ST is spreading throughout the world via immigration. According to population-based national surveys (*i.e.*, National Survey on Drug Use & Health, National Health Interview Survey), the prevalence of tobacco use for south Asians living in the US is between 7.0%–12.4% [13], with the majority of tobacco products being smokeless- either homemade (paan) or manufactured (paan masala, gutkha) [14]. 

In general, ST products contain, among other constituents, nicotine and known carcinogenic chemicals such as tobacco-specific N-nitrosamines (TSNA), benzo[a]pyrene, nitrate, cadmium, lead, asenic, nickel, and chromium [15]. A number of serious, adverse human health outcomes have been linked to ST use [9,16]. These include: periodontitis [17]; oral leukoplakia and submucous fibrosis; gastrointestinal abnormalities [18,19]; oropharyngeal, esophageal and pancreatic cancers [20,21,22]; as well as cancer of the stomach [23]. Other potential adverse health effects of ST may include toxicity of the immune-, cardiovascular-, and/or reproductive systems [16]. 

The majority of research attention on ST has focused on cancer from an epidemiological or clinical standpoint, along with usage [24,25], harm-reduction potential [26,27,28], and risk perceptions [29,30]. Limited studies using animal models have demonstrated that long-term gutkha exposure can result in reproductive toxicity including lowered spermatid/sperm count and sperm production in male mice [31]. Long-term exposure of mice to gutkha caused decreased antioxidant defense resulting in long-term inflammation in the liver and lung [32]. 

Short-term exposure studies to different ST forms in animal models are very much lacking, particularly those involving gutkha. Given the growing prevalence and global use of culturally-specific tobacco products, including gutkha, a murine study was performed to determine the toxicological implications of guthka using an exposure route relevant to the human experience. 

## 2. Materials and Methods

### 2.1. Animals

Upon arrival to NYU, 8-week-old male B_6_C_3_F_1_ mice (Charles River Laboratories, Kingston, NY, USA) were housed individually in polycarbonate cages in a temperature- and humidity-regulated room (22 °C and 55% relative humidity). Food and water were provided *ad libitum* and light and dark periods were maintained on 12-hour cycles. Mice were allowed to acclimate for two weeks prior to treatment and were 10-week-old at the initiation of exposure. All animal procedures were conducted under an animal use protocol approved by the New York University Institutional Animal Care and Use Committee (IACUC, New York, NY, USA).

### 2.2. Preparation and Exposure of Smokeless Tobacco Extract

An aqueous extract of gutkha was prepared as previously published [32] with slight modifications. Commercially available gutkha (RMD Manikchand, Pune, India) was finely powered using a mortar and pestle. Twenty grams of the powdered gutkha were dissolved in 50 mL of distilled water and incubated at 37 °C for 30 min with thorough shaking. The dissolved contents were filtered through 125 mm filter paper (Whatman) to remove larger-sized materials, and again through a 0.22 µm filter (Corning) to sterilize the recovered products. The recovered sterile solution was immediately frozen at −80 °C overnight and lyophilized for 2 days. The lyophilized extract (170 mg) was solublized fresh each day in 400 μL distilled water, pH tested, and administered orally by “painting” the buccal cavity (*i.e.*, upper and lower pallet and tongue) of each mouse. 

### 2.3. Experimental Design

**Figure 1:**
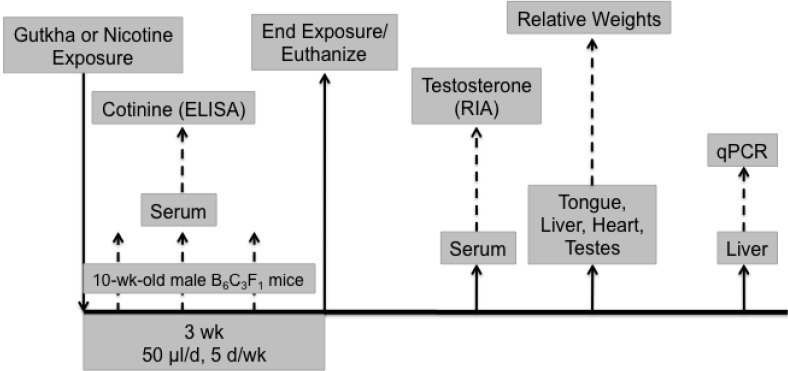
Experimental approach used to assess the toxicity of gutkha and nicotine in a mouse model.

The experimental design used for the study is shown in Figure 1. Twenty-one mice were divided randomly into three groups of seven mice each and each mouse was treated (via the oral mucosa) for 3 week (5 day/week, once/d) in the same manner with either: (1) equal volumes (50 μL) of water (control); (2) a solution of nicotine dissolved in water, containing 0.24 mg of nicotine; (3) or the gutkha solution as described above. Each mouse was weighed daily prior to treatment. Blood was collected (via tail bleed) weekly from a subset of each treatment group (3 mice/group) approximately 1 h after exposure and serum cotinine measured using an EIA kit according to the manufacturer’s instructions (OraSure Technologies, Inc., Bethlehem, PA, USA). All mice were euthanized 24 h after the last exposure by ip injection of pentobarbital (0.2 mL of Sleepaway (Fort Dodge Animal Health, Fort Dodge, IA, USA) diluted 1:10 in PBS) and blood collected from the descending aorta. Heart, testes, liver and tongue were weighed and the liver quickly frozen in liquid nitrogen and stored at −80 °C for later analysis of gene expression. 

### 2.4. RIA of Serum Testosterone

Serum testosterone was measured by radioimmunoassay (RIA) using an ^125^I assay kit and according the manufacturer’s instructions (MP Biomedicals, Costa Mesa, CA, USA). Each sample was measured in duplicate using 50 μL of serum per assay tube.

### 2.5. RNA Extraction and Real-Time PCR Analyses

The liver was used to examine mRNA expression of cytochrome P450 2A5 (CYP2A5), a major homologue of the human nicotine-metabolizing enzyme CYP2A6, using real-time PCR. Total RNA was extracted from a small piece of liver using Trizol (Life Technologies, Grand Island, NY, USA) according to the manufacturer’s directions. Following extraction, RNA samples were quantified using a NanoDrop (NanoDrop Technologies, Wilmington, DE, USA), treated with DNase I (Turbo DNA-free, Ambion, Grand Island, NY, USA) to remove any trace DNA from the sample, and then re-quantified. All RNA samples had 260/280 ratios between 1.8 and 2.0. RNA was reverse transcribed in a reaction containing 1 mg of RNA and followed the Improm-II Reverse Transcription System (Promega, Madison, WI, USA) in a volume of 25 mL, containing 5 mM MgCl_2_ and 0.5 mL RNasin (Promega), 80 mM dNTPs, and 4.8 ng/mL random hexamers (Thermo Scientific, Pittsburg, PA, USA). Complementary DNAs were used as templates for realtime PCR using Power Sybr Green Master mix (Applied Biosystems, Grand Island, NY, USA) in 10 mL reactions containing 1 mL of cDNA and 25 mM primers (using an Applied Biosystems 7900 HT Fast Realtime PCR system). Relative changes in expression were determined using the DDCt method with 18S rRNA as the housekeeping gene. A reference pool (used as a control) was made by combining equal volumes of all cDNAs. Sequences for realtime PCR were obtained from PrimerBank and primers for the 18S rRNA housekeeping gene was provided by Dr. Jason L. Blum [33]. 

### 2.6. Statistics

Except for the cotinine time course data, which were analyzed using repeated measures, data were analyzed by one-way ANOVA using IBM SPSS Statistics software (IBM, Armonk, NY, USA). When appropriate, individual groups were compared by LSD *post-hoc* testing. Means were considered different wherever *p* < 0.05. In all cases, data are presented as means ± SD.

## 3. Results

### 3.1. Serum Cotinine

**Figure 2:**
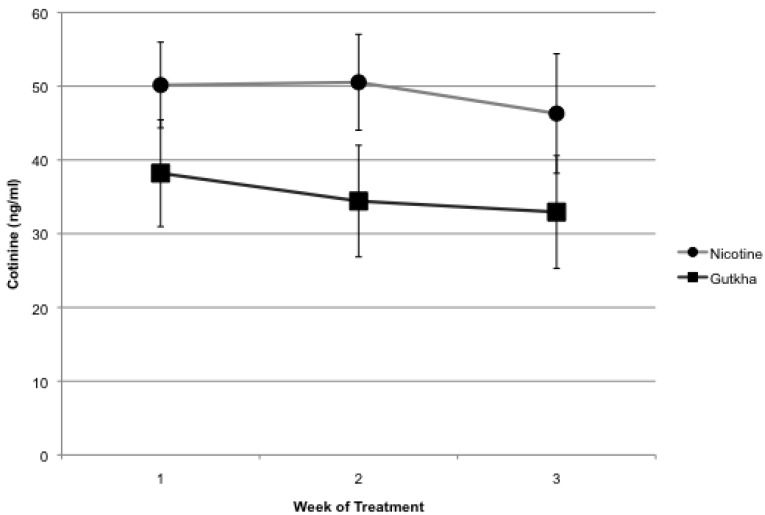
Mice treated for 3 week with either nicotine or gutkha had elevated serum cotinine levels. Cotinine levels in water-treated control mice were below assay detection limits at all time points evaluated. Data represented as means +/− standard deviation, *n =* 3 mice/group for each time point.

Serum cotinine, a major metabolite of nicotine, was measured weekly (*i.e.*, three mice/group, 1 h after treatment) for mice from each of the three treatment groups. Treatment of mice with either nicotine or gutkha yielded elevated serum cotinine levels, averaging ~49 and ~35 ng/mL, respectively, which did not change statistically over the 3 week treatment period (*p* > 0.05, Figure 2). 

Treatment of mice with nicotine averaged 50.1 (±5.8), 50.5 (±6.5), and 46.3 (±8.1) ng/mL over the course of three weeks, while in gutkha-treated mice serum cotinine averaged 38.2 (±7.2), 34.4 (±7.6), and 32.9 (±7.6) over the same time frame. Differences in serum cotinine levels between these two groups were not statistically significant over the 3 week time period period. Levels of cotinine in water-treated control mice were below assay detection limits (*i.e.*, <5 ng/mL). 

### 3.2. Body and Organ Weights

**Figure 3:**
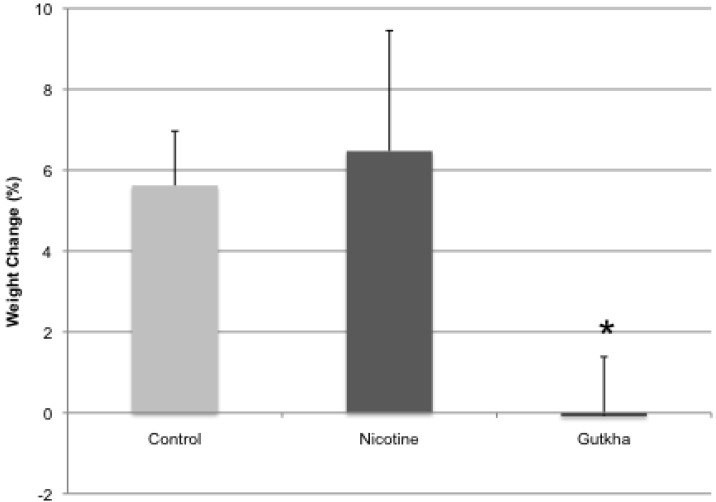
Overall body weight change after exposure for 3 week to either water, nicotine or gutkha. Body weight decreased in gutkha-exposed mice compared to controls (*p <* 0.05), but not in mice exposed to nicotine alone. *n =* 7–12 animals/group.

Note: * indicates p < 0.05 compared to control.

All mice were weighed daily before each exposure and then again immediately prior to sacrifice. When body weight (BW) on day one of treatment was compared to BW of each mouse from a given treatment group 3 weeks later on the day of sacrifice, body weight gain was significantly reduced in gutkha treated animals compared with control and the nicotine-treated group (Figure 3). No difference was observed between percent BW gain of control- and nicotine-treated group. BW of gutkha-treated mice was decreased by 0.1 (±1.3%) following treatment for 3 week, while BW of control and nicotine treated mice was increased over time by 5.6 (±1.3%) and 6.5 (±3.0%), respectively. 

**Figure 4:**
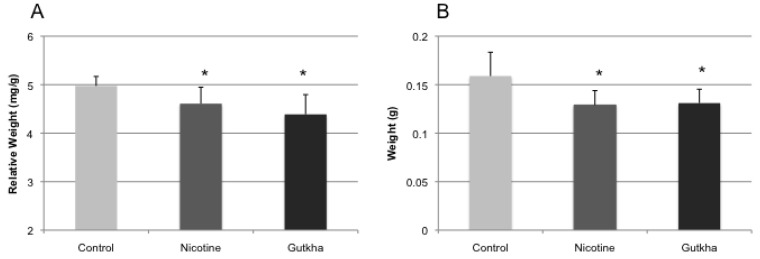
Relative (**a**) and absolute (**b**) heart weight. Both relative heart weight (organ weight/BW) and absolute heart weight was significantly decreased in both nicotine- and gutkha-exposed mice compared to controls (*p <* 0.05). Data represented as means +/− standard deviation, *n =* 7–12 mice/group.

Note: * indicates p < 0.05 compared to control.

To examine the effects of nicotine and gutkha treatment on relative organ weight (organ weight/BW), heart, liver, tongue, and testes were weighed immediately following sacrifice. Relative and absolute heart weight was significantly reduced in nicotine and gutkha-treated mice compared to their control counterparts (Figure 4a,b). No significant difference was observed in relative and absolute heart weight between the nicotine- and gutkha-treated groups. Relative heart weight averaged 5.0 (±0.2) and 4.6 (±0.3) mg/g in control- and nicotine-treated groups, respectively, while relative heart weight in gutkha-treated mice averaged 4.3 (±0.4) mg/g. Absolute heart weight averaged 0.16 (±0.02), 0.13 (±0.01), and 0.13 (±0.01) g in control-, nicotine-, and gutkha treated groups, respectively. 

**Figure 5:**
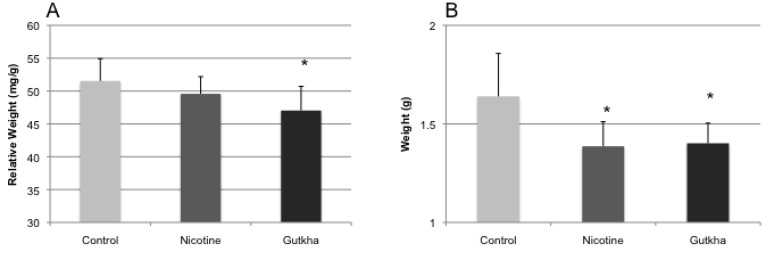
Relative (**a**) and absolute (**b**) liver weight. Relative (organ weight/BW) was significantly decreased in gutkha-exposed mice (*p <* 0.05), but not in mice exposed to nicotine alone (compared to controls). Absolute heart weight was decreased in both nicotine- and gutkha-exposed mice compared to controls (*p <* 0.05). Data represented as means +/− standard deviation, *n =* 7–12 mice/group.

Note: * indicates p < 0.05 compared to control.

While relative liver weight of nicotine-treated mice was slightly lower than that of control values (albeit, not significantly), a significant reduction in relative liver weight was observed in gutkha-treated mice when compared to the control or nicotine-treated groups (Figure 5a). No significant differences in relative liver weight were observed between control and nicotine-treated mice. Relative liver weight averaged 51.5 (±3.4), 49.6 (±2.6), and 47.0 (±3.7) mg/g, respectively, in water-, nicotine- and gutkha-treated animals. Absolute liver weights were significantly reduced in the nicotine- and gutkha-treated groups compared to control (Figure 5b). Absolute liver weights averaged 1.64 ± 0.22, 1.39 ± 0.12, and 1.40 ± 0.10 g in the control-, nicotine-, and gutkha-treated groups, respectively.

Normalized and absolute weights of the tongue and testes were not different between any of the groups. Relative tongue weight averaged 3.3 ± 1.3, 2.9 ± 0.48, and 3.1 ± 0.39 mg/g and relative testis weight averaged 7.3 ± 1.3, 7.8 ± 0.10, and 7.7 ± 1.3 mg/g, respectively, in water-, nicotine- and gutkha-treated animals. No morphological changes were observed in the tongue in any of the treatment groups. 

### 3.3. Serum Testosterone

**Figure 6:**
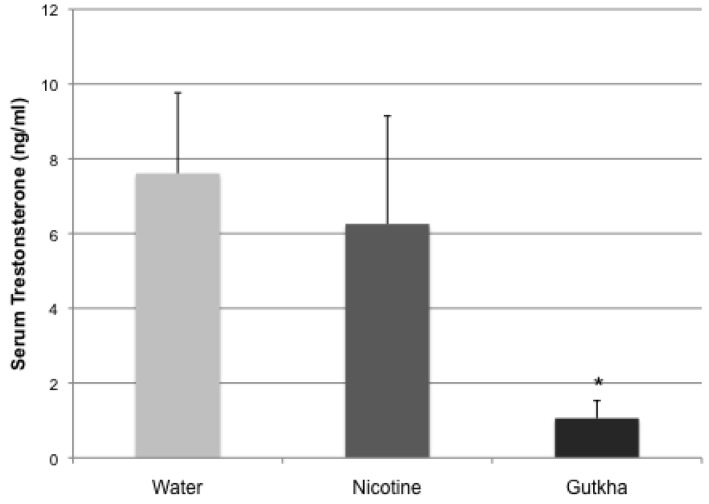
Serum testosterone levels were significantly decreased in gutkha-exposed mice (*p <* 0.05), but not in mice exposed to nicotine alone compared to controls. Data represented as means +/− standard deviation, *n =* 7 mice/group.

Note: * indicates p < 0.05 compared to control.

Circulating testosterone, measured by RIA, was significantly decreased (compared to control) in gutkha-treated mice (Figure 6). In contrast, no significant differences (compared to control) were observed in the nicotine-treated group (*p* > 0.05). Serum testosterone levels averaged 7.61 ± 2.16, 6.26 ± 2.89, and 1.06 ± 0.47 ng/mL, respectively, in the control, nicotine- and gutkha-treated groups. 

### 3.4. Liver CYP2A5 Expression

**Figure 7:**
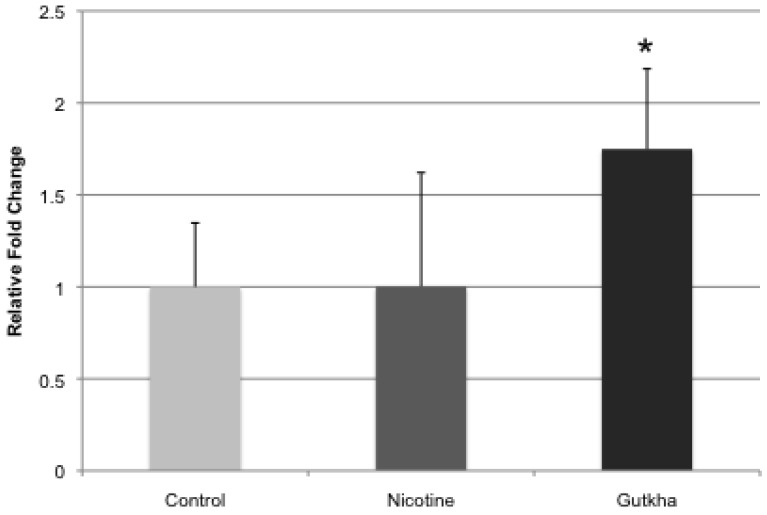
Relative fold-change in gene expression of CYP2A5 in the liver was significantly increased in gutkha-treated mice (*p <* 0.05), but not in mice exposed to nicotine alone compared to controls. Data are represented as means +/− standard deviation, *n =* 7 mice/group.

Note: * indicates p < 0.05 compared to control.

CYP2A5 expression in the liver, measured by real-time PCR, was significantly increased (approximately two-fold) in the gutkha-treated group with no effect on expression observed in the nicotine-treated mice (compared to control, Figure 7). 

Relative fold change averaged 1.0 (±0.35) and 1.75 (±0.44) in the nicotine- and gutkha-treated groups, respectively. 

## 4. Discussion and Conclusions

These studies utilized a mouse model and a novel oral mucosal exposure scenario to assess toxicity of a globally-used ST product in adult male mice. Results here demonstrated that repeated, short-term (three week) oral exposure to gutkha produced a variety of adverse morphological and metabolic changes including reduced body weight gain, lowered heart and liver weight, decreased circulating testosterone levels, and increased expression of hepatic CYP2A5. Such effects suggest that use of gutkha, even for a relatively short time period, produces organ system toxicity and alters critical circulating hormone and enzyme levels. As use of ST products in general is increasing worldwide, particularly among adolescents and young adult males [11], these findings have major public health implications.

A strength of this study includes the innovative ST oral delivery system. While *in vitro* studies have been performed examining the toxicological implications of a variety of ST products [34,35,36] and *in vivo* animal systems have examined the effects of ST via food or drinking water [31,37,38], oral mucosal absorption of ST, that most closely reflects the human ST experience, has not yet been studied. Another strength of this study is the inclusion of nicotine alone into the experimental design (at approximately the same circulating cotinine level as that produced by gutkha) that serves to both gauge effects of gutkha compared to a known tobacco toxicant and aids in identifying key components that may contribute to the observed effects. Although pH of the gutkha solution was higher than that of the nicotine solution (8.0 *vs*. 6.5), and nicotine at an alkaline pH has been shown to be more readily absorbed through the mucous membrane than acid/neutral environments [39], cotinine levels were similar in both treatment groups, implying that both sets of mice received the same nicotine dose.

A study by Kumari *et al*. [31] demonstrated that chronic exposure of mice for six months to 3% pan masala with tobacco (a South Asian product similar to gutkha) in the diet produced adverse reproductive outcomes including reductions in spermatid count, mature sperm count, and sperm production. The investigators also reported testicular damage and abnormal sperm head shape, as well as a considerable decline in testicular 17β-hydroxysteroid dehydrogenase (17β-HSD) activity; reduced activity of 17β-HSD in the pan masala-treated mice indicates impairment of steroidogenesis. Such a change in 17β-HSD activity can lead ultimately to reduced circulating testosterone levels [31]. As testosterone is required for the attachment/adherence of different generations of germ cells in seminiferous tubules, reduced levels can lead to detachment of germ cells from seminiferous tubule epithelium, potentially leading to infertility [40]. Studies here demonstrate that short-term gutkha exposure can lead to decreased testosterone, that could potentially impact male fertility.

In contrast to that seen in our short-term studies, chronic exposure of mice to pan masala in the diet produced a significant decline in testis weight [31,41]. Conflicting data between studies may be due to differences in exposure duration, exposure route, and/or overall dose, as mice were exposed here to a lower concentration by the oral mucosal route and for a shorter time period. As both testes were weighed together for a given animal in our studies, the effect of gutkha on bilateral distribution of the testes also could not be accounted for. Few clinical studies have examined semen quality in men who use any form of ST. However, a study by Said *et al*. [42] reported a significant decrease in semen quality (*i.e.*, reduced sperm count, motility, viability and altered morphology) associated with high (*i.e.*, >6 times per day), moderate (*i.e.*, 3–6 times per day) and mild (*i.e.*, <3 times per day) use of any form of ST in men undergoing infertility evaluation. All ST users in this study had a history of use of four to ten years. These findings are in contrast to earlier studies by Dikshit *et al*. [43] who reported no significant difference in sperm parameters between ST users (of any form) and non-users. The former study included a larger cohort of patients, and also compared patients according to their rate of consumption, likely providing greater power for discerning statistical differences.

It has been reported that both nicotine and its longer-lived metabolite, cotinine, can produce dose-dependent inhibition of LH-stimulated testosterone production in isolated mouse Leydig cells [44]. However, in studies here, nicotine had no effect on circulating testosterone levels compared to control, suggesting that effects of nicotine on testosterone levels are more complex *in vivo*. Overall, the results here suggest that under similar exposure conditions and relatively equivalent internal cotinine levels, gutkha acts more dramatically than nicotine alone to adversely affect circulating testosterone levels.

It is widely accepted that cigarette smoking leads to loss of appetite with subsequent weight loss [45,46,47], and cessation of cigarette smoking usually results in the opposite effect [48,49,50]. This effect has also been observed in rats following a 4-week cigarette smoke exposure [51]. Little is known, however, about the effect of ST, and in particular gutkha, on body weight changes. In the current study body weight gain was significantly suppressed in mice treated with gutkha compared to that seen in control mice. Because food intake and feeding patterns were not accounted for, weight loss could have been due to a reduction in appetite/food intake. However, treatment with nicotine failed to alter body weight gain, suggesting that something other than nicotine was responsible for the gutkha-induced change in body weight.

Relative and absolute liver weight was significantly decreased in mice treated with gutkha (compared to their control counterparts), with no relative liver weight changes seen in the nicotine-treated groups. Avti *et al*. [32] noted mild to moderate inflammatory changes in the liver of rats following a 32-week exposure to an aqueous extract of gutkha, orally administered by daily gavage at a dose of 96 mg/kg BW (in a volume of 300 μL). The same investigators also noted a significant decrease in liver antioxidant status, with gutkha causing decreased glutathione (GSH) levels and a reduction in the activities of superoxide dismutase (SOD), catalase (CAT), and glutathione peroxidase. Such alterations could lead to increased oxidative stress, ultimately resulting in cell damage, eventual cell death and in turn to a reduction in liver weight. As with testosterone levels, treatment with nicotine alone had less of an effect on liver weight than gutkha exposure, suggesting that hepatic effects may be due to gutkha components other than nicotine or those in combination with nicotine. 

Hepatic CYP2A5 mRNA expression was significantly increased in gutkha-treated mice, but not in mice treated with nicotine alone. It is well known that CYP2A5, the mouse homologue for human CYP2A6, plays a major role in metabolism and clearance of nicotine in the mouse [52]. In addition to nicotine metabolism, CYP2A5 is also responsible for the metabolism of structurally-similar carcinogenic nitrosamines [53], many of which are found in ST products, including gutkha [54,55]. One possible explanation for the increased expression of CYP2A5 in the gutkha-treated mice could be absorbance of additional tobacco-specific nitrosamines, which would not be the case with nicotine treatment alone. In addition, testosterone is also a substrate of CYP2A5 demonstrating high affinity for the enzyme [56,57]. Gutkha-induced decreases in circulating testosterone levels could thus be explained, in part, by the observed increase in CYP2A5 expression. In general, exposure to carcinogenic nitrosamines and nicotine in gutkha could have caused increased hepatic expression of CYP2A5, which in-turn facilitated a decrease in circulating testosterone levels.

Taken together, these findings demonstrate that short-term exposure of mice to guhtka leads to reduced levels of circulating testosterone and normal body weight gain, as well as reduced liver weight. Further investigation is needed to determine the relationship between these outcomes. As the same effects were not observed following treatment with nicotine alone, gutkha and (possibly) other chemically similar ST products may prove more toxic in some regards. These toxicological studies demonstrate that ST products, thought by many to be “safer,” than cigarettes, and lead to a variety of long-term adverse health outcomes. Future studies are needed to determine the mechanism(s) that underlie the observed effects, as well as to identify particular gutkha component(s) responsible for the observed effects in addition to their mode of action following both long term and short term exposure.
